# Prognostic Implications of Pyroptosis-Related Gene Signatures in Lung Squamous Cell Carcinoma

**DOI:** 10.3389/fphar.2022.806995

**Published:** 2022-01-27

**Authors:** Tingting Li, Huanqing Liu, Chunsheng Dong, Jun Lyu

**Affiliations:** ^1^ Department of Pharmacy, Xi’an Chest Hospital, Xi’an, China; ^2^ Department of Clinical Research, The First Affiliated Hospital of Jinan University, Guangzhou, China; ^3^ School of Computer Science, Shaanxi Normal University, Xi’an, China

**Keywords:** pyroptosis, lung squamous cell carcinoma, prognosis, TCGA, immune microenvironment

## Abstract

**Background:** Lung squamous cell carcinoma (LUSC) has been a highly malignant tumor with very poor prognosis. It is confirmed that pyroptosis refers to the deaths of cells in a programmed and inflammatory manner. Nevertheless, the correlation between expression of genes related with pyroptosis and their prognosis remains uncertain in LUSC.

**Methods:** Utilization of The Cancer Genome Atlas (TCGA) cohort has been done for evaluating the prognostics of pyroptosis-related genes for survival and constructing a signature with multiple genes. The least absolute shrinkage and selection operator (LASSO) Cox regression was performed for establishing such pyroptosis-related gene signature.

**Results:** Eventually, identification of 28 genes in relation to pyroptosis was made in LUSC and healthy lung tissues. Upon the basis of these differentially-expressed genes (DEGs), the patients of LUSC can be divided into two subtypes. Nine gene signatures were established using LASSO. The surviving rate for low-risk group was apparently greater in contrast with the high-risk group (*p* < .001). According to our finding, risk score worked as an independent predictive factor of OS among LUSC sufferers in combination with clinical characteristics. In line with Gene Ontology (GO) and Kyoto Encyclopedia of Genes and Genomes (KEGG) analyses, the enrichment of immunity-related genes and decreasing immunity status among the high-risk group.

**Conclusion:** Genes in relation with pyroptosis played an essential role in tumor immunity, which is capable of predicting the prognosis for LUSCs.

## 1 Introduction

Pulmonary carcinoma, the most serious malignant tumor, has been emphasized as a predominant reason for carcinoma death occurring in both developed and developing nations in the world (Mattiuzzi and Lippi, 2020). Current statistics have shown that 2.21 million patients were diagnosed with lung carcinoma in 2020; moreover, approximately 1.8 million patients had died from lung carcinoma, ranking as the highest mortality rate of all cancers (Ferlay et al., 2020). Despite radiation and targeted therapies, the survival from lung cancer has not clearly improved, the overall 5 years surviving rate remains under 20% (Siegel et al., 2018). Such phenomenon has seriously impacted human health, and lung cancer has attracted the public attention to health. As a common histologic subtype of lung carcinoma, LUSC is often not promptly diagnosed clinically and has inherent resistance to radiation and chemotherapy due to its early symptoms is not typical. The lack of appropriate targeted drugs leads to the poorer prognosis of LUSC than that of lung adenocarcinoma (Hirsch et al., 2017). Up to now, prognosis prediction of LUSC still mainly relies on pathological diagnosis and tumor stage system. However, traditional approaches are incapable of accurately evaluating prognoses for LUSC sufferers. Establishing a novel and reliable prognostic model is important to improve the quality, prognosis and OS of patients with LUSC.

Research on programmed cell death has recently attracted considerable attention. Pyroptosis is a programmed cell death associated with the release of an inflammatory molecule in response to stimuli, such as a pathogenic microorganism or chemotherapeutic agent, during which the cell membrane is perforated by the Gasdermin (GSDM) family protein (Galluzzi et al., 2018). Pyroptosis’s function in tumor has caused wide concern. Pyroptosis, as a new cellular death type, has exerted both positive and negative effects concerning pathogenesis and treatment of tumors. For one thing, different stimulating factors induce cell pyroptosis to form an inflammatory microenvironment during tumorigenesis, making normal cells transform into tumor cells (Karki and Kanneganti, 2019). For another, pyroptosis has inhibitory function in preventing tumors from occurring and developing (Nagarajan et al., 2019). Explorations have shown the relation amid the potent pro-inflammatory effect of pyroptosis and the regulatory effect of tumor immunity microenvironment. Gasdermin D (GSDMD) expression deficiency was in the company of a sharp reduction in quantity and activity of CD8 + T lymphocytes (Xi et al., 2019). Pizato et al. (Pizato et al., 2018) have reported that pyroptosis was closely associated with breast cancer cell death and further contributes to the improvement of breast cancer treatment. Some researchers have revealed the positive correlation between p53 and pyroptosis in NSCLC tissues, and p53-induced pyroptosis could significantly inhibit tumor growth and improve symptoms and survival of NSCLC to a certain extent (Zhang et al., 2019).

Pyroptosis has a significant function in the development of tumor and antitumor processes, such as liver cancer, breast cancer and stomach cancer (Saeki et al., 2009; Wang et al., 2013; Hergueta-Redondo et al., 2014). Ye et al. (Ye et al., 2021) has demonstrated that pyroptosis-related genes had a significant function in predicting prognosis of ovarian cancer, but few studies have focused on its specific functions in LUSC. Therefore, we studied the expression levels of pyroptosis-associated genes in healthy lung and LUSC tissues in a systematical manner, for exploring the prognosis of these genes as well as investigating the correlation of pyroptosis with the tumor immune microenvironment.

## 2 Methods

### 2.1 Pyroptosis-Related Gene Datasets and Patient Samples

Extraction of 33 pyroptosis-related genes out of prior reviews was conducted (Man and Kanneganti, 2015; Wang and Yin, 2017; Karki and Kanneganti, 2019; Xia et al., 2019; Ye et al., 2021). Download of the RNA-seq and patients with LUSC was done out of The Cancer Genome Atlas (TCGA) (https://portal.gdc.cancer.gov/repository). 507 RNA-seq (42 normal and 465 tumor) samples were obtained. With utilization of the “limma” package, DEGs possessing a *p* value < .05 were identified.

### 2.2 Establishment and Verification of the Pyroptosis-Associated Gene Prognosis Model

In order to evaluate the prognosis of pyroptosis-associated genes, utilization of Cox regression analyses was further done for evaluating the correlation between surviving state and each gene in the TCGA cohort.

For preventing negligence, the truncated *p* value had been adjusted to 0.05 and nine surviving-related genes were screened out for additional analyses. Then, LASSO Cox regression model (R-package “glmnet”) was used for narrowing the scope of alternative genes and establishing a prognosis model. Retention of nine genes together with their coefficients was conducted; while, the penalty parameter *λ* was determined according to the minimal criterion. Centralized standardization was carried out for TCGA expression data, and utilization of the scale function in R was done for calculating the risk score. The risk score formula is shown here: risk score = (coefficient mRNA1 × expression of mRNA1) + (coefficient mRNA2 × expression of mRNA2) +⋯ + (coefficient mRNAn × expression mRNAn). In line with the median risk score, division of LUSC sufferers was performed as low-risk subgroup and high-risk subgroup; and the OS time between two subgroups was analyzed and compared by Kaplan-Meier analysis. PCA analyses were conducted upon the basis of the characteristics of nine genes. Plotting of a time-dependent receiver operating characteristic (ROC) curve should be accomplished for predicting the precision of the prognosis indicators for LUSC sufferers.

Functional enrichment analyses of the DEGs amid the low-risk and high-risk groups.

Division of LUSC sufferers in the TCGA cohort was performed as two subgroups upon the basis of median risk score. According to the certain standard |log_2_ FC|≥ 1 and FDR <0.05, the DEGs between the two groups were screened. On this basis, utilization of “clusterProfiler” package was conducted for analyzing Gene Ontology Analysis (GO) and Kyoto Encyclopedia of Genes and Genomes (KEGG). “gsva” package was employed for performing single-sample gene set enrichment analyses (ssGSEA), calculating the score of infiltrated immunity cells and evaluating the activity of immunity-associated pathways.

### 2.3 Statistics Analyses

Single-factor ANOVA had been performed for comparing gene expression levels amid healthy tissues and LUSC tissues, and with Pearson χ^2^ test, a comparison of taxonomical variables was carried out. For comparing the patients’ OS rates between the subgroups, Kaplan Meier methodology and two side log-rank test were utilized. Univariate and multivariate Cox regression models had been applied for assessing the independent prognostics for the risk model. With utilization of Mann Whitney test, a comparison of the immunity cellular infiltration and immunity pathway activation was performed amid the two groups. Statistics analysis was performed using R software (V4.0.2).

### 3 Resluts

#### 3.1.1 Authentication of DEGs Between the Healthy and Tumor Tissues

**FIGURE 1:**
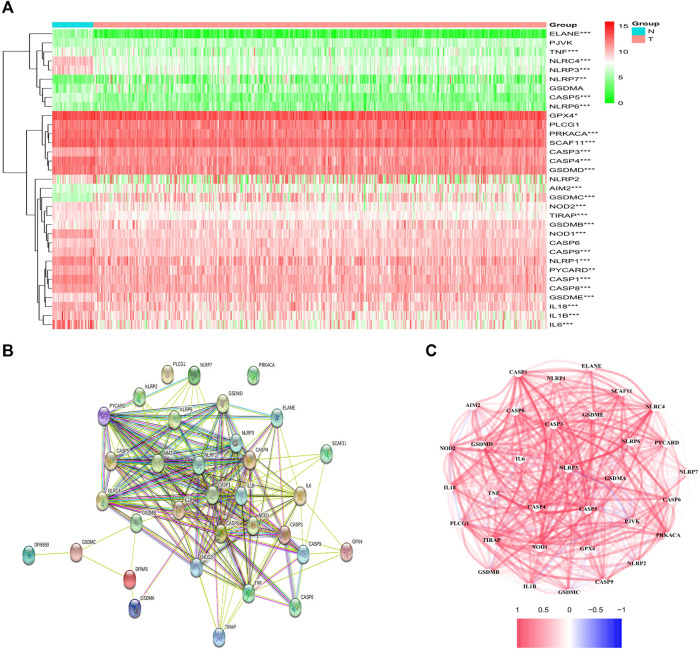
Expression and interaction of DEGs. **(A)** Heatmap of DEGs amid normal tissue (N, bright blue) and tumor tissue (T, red) (green: low expression level; Red: high expression levels). **p < .01; ***p < .001. **(B)** PPI network showed the interaction of DEGs (interaction score = 0.4). **(C)** DEGs correlation network (red line: positively correlated; blue line: negatively correlated. The depth of the color gives a reflection on the strength of the correlation).

From the TCGA data of 42 healthy and 465 tumor tissues, a comparison of 33 pyroptosis-associated gene expression levels was made and 28 DEGs were identified. Among these DEGs, 22 genes were downregulated (i.e., *ELANE*, *IL6*, *NLRC4*, *NLRP3*, *CASP5*, *IL1B*, *NOD1*, *CASP1*, *CASP4*, *TNF*, *NLRP1*, *TIRAP*, *NLRP6*, *IL18*, *GSDMD*, *PRKACA*, *NOD2*, *CASP9*, *CASP8*, *SCAF11*, *PYCARD,* and *GPX4*), while upregulation happened to six other genes in the tumor group (i.e., *CASP3*, *GSDMB*, *GSDME*, *AIM2*, *NLRP7*, and *GSDMC*). The RNA levels of the genes are shown in Figure 1A. For further exploring the interacted status of pyroptosis-associated genes, a Protein Interaction (PPI) analysis was performed, as indicated in Figure 1B. Through setting of the minimal required interactive score as 0.4 (medium confidence) for PPI analysis, *CASP1*, *PYCARD*, *NLRC4*, *NLRP1, CASP5, NLRP3*, *CASP8* and *AIM2* were identified as hub genes according to our determination. Apart from *CASP1* gene, all the others were DEGs amid healthy tissue and tumor tissue. The correlated network comprising each gene in relation with pyroptosis is shown in Figure 1C.

### 3.2 Tumor Classification Upon the Basis of the DEGs

**FIGURE 2:**
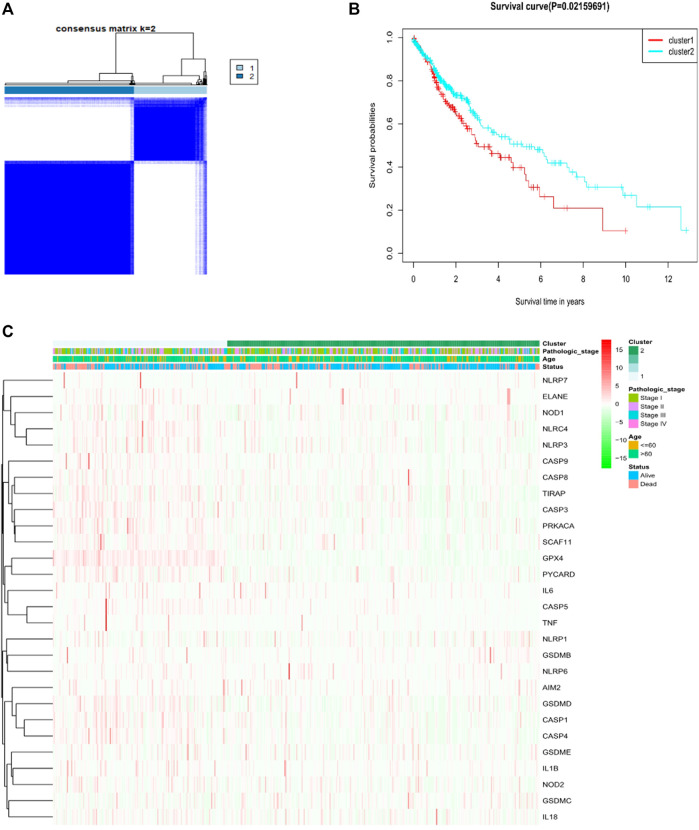
Tumor classification upon the basis of pyroptosis-related DEGs. **(A)** Total of 491 LUSC patients were clustered in line with the consensus clustering matrix **(B)** Heatmap and clinicopathological features of the two clusters according to these DEGs (StageⅠ, StageⅡ, StageⅢ and Stage Ⅳ are the degree of tumour differentiation). **(C)** Kaplan–Meier OS curves for the two clusters.

For investigating the relationship amid the expression of the 28 pyroptosis-associated DEGs and LUSC subtypes, consistent cluster analyses on total 491 LUAD sufferers in the TCGA cohort were done. Through elevating the clustering variable (k) amid 2 and 10, the greatest correlation within group and low correlation amid groups were revealed when k = 2, suggesting the practicability of dividing the patients as two clusters according to 28 pyroptosis-related DEGs (Figure 2A). Protein expression profiles and clinical characteristics included heat maps of the degree of tumor differentiation (stages I–IV), age (≤60 years or >60 years), and surviving state (survival or death). However, we found little difference in the clinical characteristics amid two clusters (Figure 2B). Overall survival time (OS) had been found amid the two groups (*p* = 0.022, Figure 2C).

### 3.3 Construction of a Prognostics Gene Model

**FIGURE 3:**
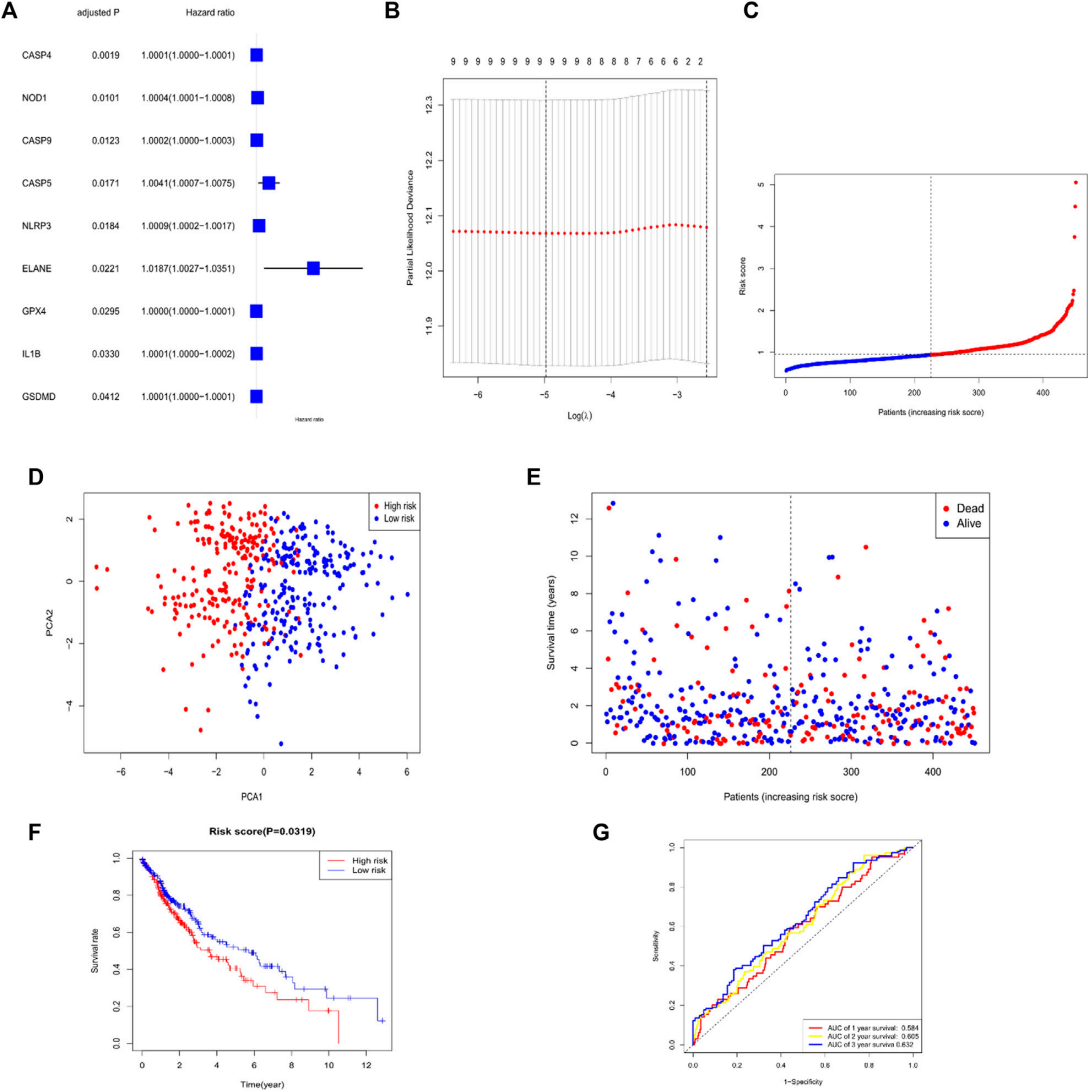
Establishment of risk signature in the TCGA cohort. **(A)** Univariate Cox regression analysis was carried out for the OS of all the pyroptosis-related genes, nine of which were *p* < .05 **(B)** LASSO performed regression analysis on nine OS-associated genes. **(C)** Patients distribution upon the basis of risk score **(D)** PCA plot for LUSC upon the basis of risk score. **(E)** Surviving state of all patients (low-risk population: dashed line left; High-risk group: right of dotted line). **(F)** Kaplan–Meier curves for the OS of patients in the high- and low-risk group **(G)** ROC curves manifested the prediction effect of the risk score.

For investigating the effect of the pyroptosis-associated DEGs on LUSC prognosis, Cox univariate analysis (Figure 3A) had been done. Through utilization of LASSO Cox regression model, we selected of genes with the greatest prediction as prognosis indexes. At the time that the median of the sum of squares of residuals was the smallest, *λ* was selected. A nine-gene signature was done in line with the optimal *λ* value (Figure 3B). *CASP4*, *NOD1*, *CASP9*, *CASP5*, *NLRP3*, *ELANE*, *GPX4*, *IL1B*, and *GSDMD* were identified as prognostic factors for LUSC. Thus, the formula for our model was as follows: Risk Score =(2.154e−05 × expression *CASP4*) + (1.740e−04 × expression *NOD1*) + (9.836e−05 × expression *CASP9*) + (1.433e−03 × expression *CASP5*) + (9.719e−05 × expression *NLRP3*) + (1.006e−02 × expression *ELANE*) + (1.652e−05 × expression *GPX4*) + (7.975e−05 × expression *IL1B*) + (8.574e−06 × expression *GSDMD*). In addition, calculation of the risk score for all patients was done in the study cohort. Then, division of this cohort was performed as high-risk group and low-risk group, employing the median risk score as the cut-off value (Figure 3C). As shown in Figure 3D, the sufferers possessing various risks could be divided into the two groups by principal component analysis (PCA). More deaths and less surviving times were seen in the high-risk group versus the low-risk group (Figure 3E). Significant difference was in OS time amid the low-risk group and high-risk group (*p* < .05, Figure 3F). The ROC curves were also applied for investigating whether the expression patterns of the pyroptosis-associated DEGs could predict the prognosis of LUSC. The AUC values for the 1 year, 2 years, and 3 years survival were 0.584, 0.605, and 0.632, respectively (Figure 3G).

### 3.4 Independent Prognostics for the Risk Model

**FIGURE 4:**
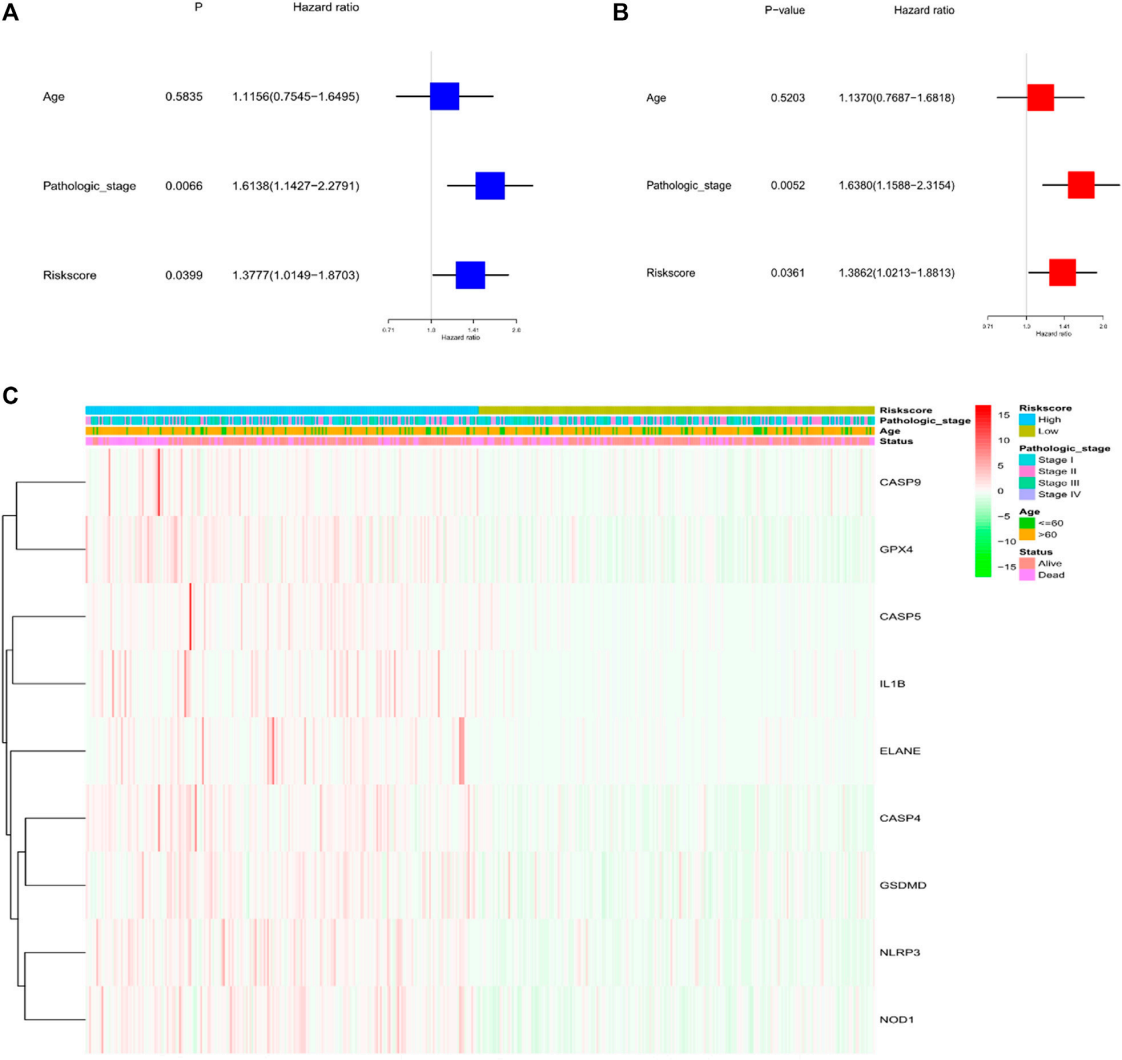
Univariate and multivariate Cox regression analysis for the risk score. **(A)** Univariate analysis for the TCGA cohort (pathologic stage: the degree of tumour differentiation, Ⅰ to Ⅳ). **(B)** Multivariate analysis for the TCGA cohort. **(C)** Heatmap (green: low expression; red: high expression) to connect clinical pathologic characteristics with the risk groups.

Through univariate and multivariate Cox analyses, the possibility of using risk factors as an independent prognosis factor was known. Based on the characteristics of the nine pyroptosis-related genes, risk score (HR = 1.3777, 95% CI: 1.0149–1.8703, *p* < .05) was shown as an independent prognostics for LUSC (Figure 4A). Multivariate analyses revealed the possibility of using risk score as a prognostic factor after adjusting for other confounders (HR = 1.3862, 95% CI: 1.0213–1.8813, *p* < .05; Figure 4B). Additionally, a heat map of the clinical characteristics of the TCGA cohort were generated, as shown in Figure 4C, finding that the distribution in the age and surviving status of patients was different amid the low-risk subgroup and high-risk subgroup (*p* < .05).

### 3.5 Functional Analyses Upon the Basis of the Risk Model

**FIGURE 5:**
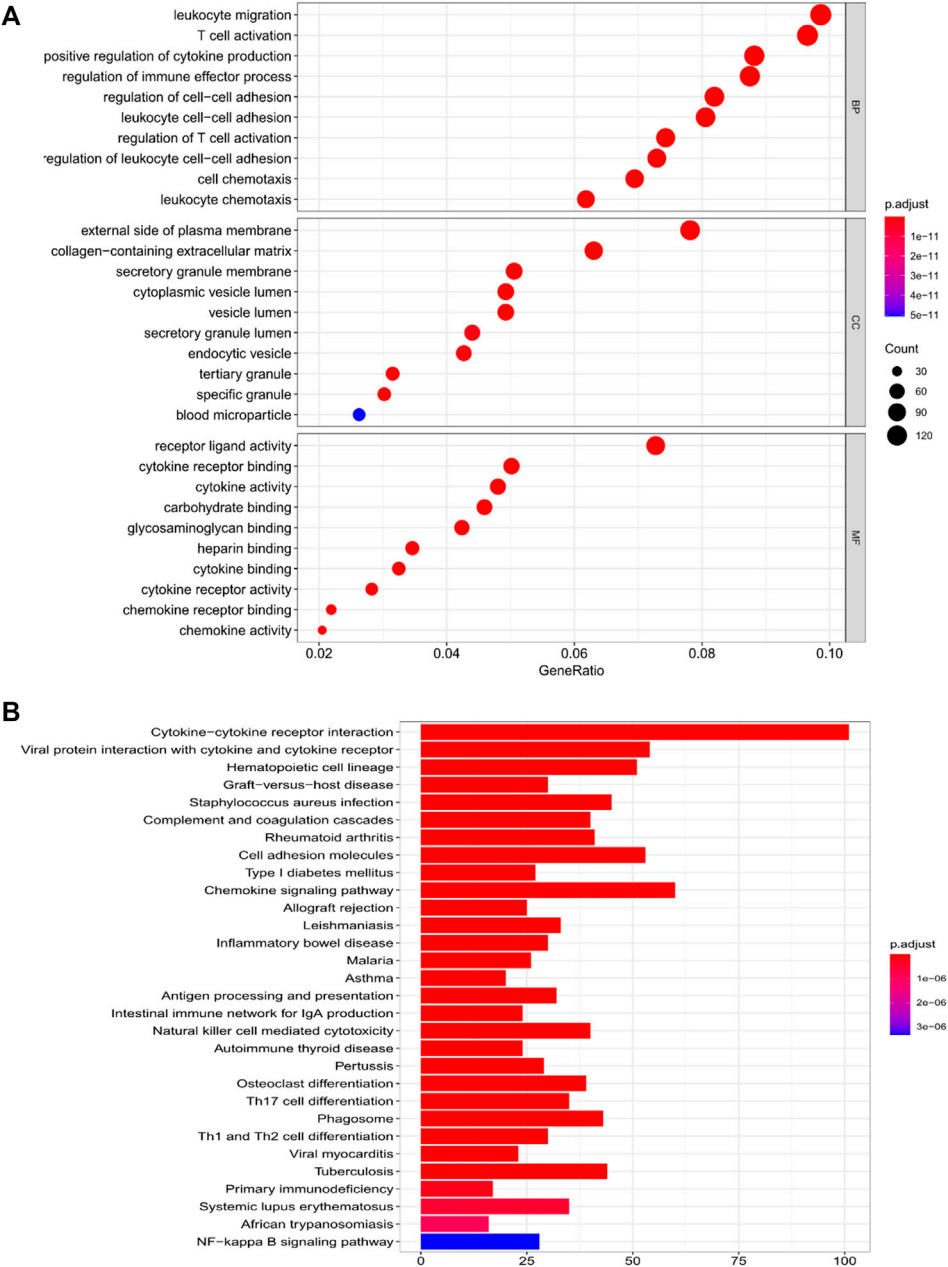
Functional analysis of genes that differed amid the two risk groups in the TCGA cohort. **(A)** Bubble diagram of GO enrichment (the larger the bubble is, the greater number of genes are enriched; the deeper the red depth is, the obviously greater the difference is). **(B)** KEGG pathway Barplot (the longer the bar is, the greater number of genes are enriched, and the deeper the red is, the obviously greater the difference is).

We used limma R package for further exploring the classification of risk model subgroup differences between the gene function and pathways. In the TCGA cohort, altogether 1,637 DEGs were found in the low-risk group and in-risk group, among which upregulation was seen in 1,628 genes and downregulation was seen in nine genes. (Supplementary Table S1). GO enrichment analysis and KEGG pathway analysis had been conducted among DEGs. Results showed main association of DEGs with leukocyte migration, regulation of immune effector process, and cytokine-cytokine receptor interaction (Figures 5A,B).

### 3.6 Combination of the Immune Activity in the Subgroups

**FIGURE 6:**
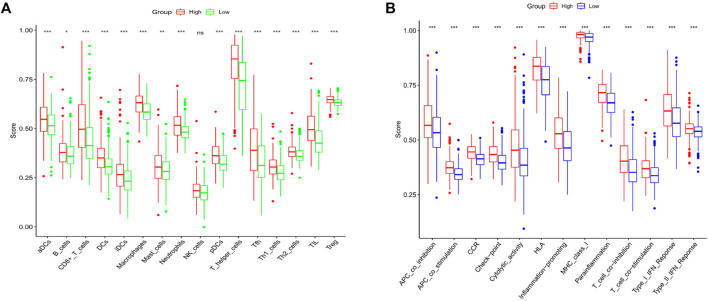
Contrast of ssGSEA scores of immune cells with immune pathways. **(A)** Contrast was made of the enrichment scores for 16 immunity cells in the low (green box) group and the high risk (red box) group in the TCGA cohort. **(B)** Contrast of enrichment scores of 13 immune-associated pathways in low (blue box) and high risk (red box) groups in the TCGA cohort.

Upon the basis of functionality analysis, ssGSEA was further performed. The enrichment fractions of 16 immune cells and the activities of 13 immune-associated pathways were compared aimed the low-risk and high-risk populations in the TCGA cohort. According to Figure 6A, compared with the low-risk subgroup, besides the nature killer (NK) cells, the infiltrative level of immunocytes in the high-risk subgroup was generally lower. Additionally, all 13 immune-associated pathways had less activity in high-risk group versus low-risk group (Figure 6B).

## 4 Discussion

Globally, about 85% of pulmonary carcinoma patients have been confirmed with NSCLC, most of which are hard to cure because of complicated progressive diseases. Chemotherapy based on medication may provide a modest extension of survival for those sufferers. However, the efficacy of current treatments appears to be stagnating without apparent improvement in response rates or median surviving status (Schiller et al., 2002; Scagliotti et al., 2008; Drilon et al., 2012). Numerous studies have been conducted on LUSC, but this cancer is associated with poorer clinical outcomes compared with lung adenocarcinoma (Hirsch et al., 2017).

Pyroptosis, a manner of cellular deaths in a programmed and inflammatory form, causes cellular swelling, plasma membrane dissolution, chromatin rupture and release of the proinflammatory substances in cells. Activated pyroptosis causes the release of inflammatory mediators, contributing to the development and progression of carcinoma (Hu et al., 2010). Many experiments have been performed to confirm the role of pyroptosis in carcinogenesis, such as hepatic cellular cancer, breast cancer, and gastric cancer (Saeki et al., 2009; Wang et al., 2013; Hergueta-Redondo et al., 2014). The manner by which the genes associated with pyroptosis interact with each other in LUSC and whether they are related to patient survival are uncertain.

### 4.1 Nine Pyroptosis-Related Genes Predicted the Prognosis of LUSC

We identified a trait with nine pyroptosis-related genes (i.e., *GSDMD*, *CASP4*, *CASP5*, *CASP9*, *ELANE*, *NOD1*, *NLRP3*, *GPX4*, and *IL1B*) and found that these genes had the function of predicting OS in LUSC patients.

*GSDMD* belongs to a structurally and evolutionarily conserved superfamily of GSDM proteins (Saeki et al., 2009) and has been identified as the key executioner of pyroptosis (Shi et al., 2017). The homologous member of the GSDM family (GSDMA-C, DFNA5) was found in reports to play a potential role in various tumors (Gao et al., 2018). Low expression was found in GSDMD in GC cell lines and models, additionally explorations have shown that down-regulation of GSDMD is capable of regulating the expression of cellular cycle-related proteins and promote tumor cell growth (Qiu et al., 2017). Gao et al. observed the overexpression of *GSDMD* in LUAD (Gao et al., 2018), suggesting that *GSDMD* might have a special function in various cancers. Our exploration found *GSDMD* appeared as an oncogene, because it was upregulated in the tumor tissue. However, it helped to prolong patients’ surviving status because of its enrichment in the low-risk group. In consideration of the limitation in information available from LUSC and the conflicts in outcomes for various tumors, the outcomes on GSDMD offer several opinions on deeper research.

Caspases (cysteine-aspartic proteases) are proteolytic enzymes that are used primarily to control cell death and inflammation. Several caspases have been found in mammals, such as human *CASP1*, *CASP4*, *CASP5*, and *CASP12*, which were identified to be essential mediators for innate immunity responses (Martinon et al., 2004). *CASP4* and *CASP5* are human homologous genes of mouse *CASP11* (Shi et al., 2015; Zhao et al., 2018). Many conclusions about immune function of *CASP4* and *CASP5* have been based on the studies of *CASP11* in mice. *CASP4* has been shown to be directly involved in LPS sensing and served as a key factor in pyroptosis and *CASP1*-mediated IL-1*β* production in LPS-transfected human monocytes (Baker et al., 2015). *CASP4* gene silencing was found to protect THP-1 and U937 monocytes from cytoplasmic LPS-induced pyroptosis (Shi et al., 2014). *CASP5* has a synergistic effect with *CASP4.* Interestingly, *CASP4*, *CASP5*, and *CASP11* can process *GSDMD* independently of *NLRP3* and its adaptor protein ASC (He et al., 2015). By contrast, *CASP9* has not been directly associated with inflammatory responses (Galluzzi et al., 2016). However, Abe et al. (Abe and Morrell, 2016) reported that *CASP9* inhibitors significantly inhibited PI+/Annexin V cell pyroptosis, and its mechanism needs further exploration. These three screened caspase genes, *CASP4*, *CASP5* and *CASP9*, also have a significant function in antitumor. Qiao et al. (Qiao et al., 2019) demonstrated that a-NETA has induction effect upon pyroptosis of epithelial ovarian carcinoma cells through the GSDMD/caspase-4 pathway. A previous study has identified tumor-associated *CASP4* as a new diagnosis, prediction and prognosis biomarker for NSCLC patients (Terlizzi et al., 2018), nevertheless, the relation of the *CASP4*-mediated pyroptosis with LUSC development is still unknown. At the same time, according to our findings, high caspase-4 expression had association with inferior surviving prognosis, which might become the outcomes of its negatively regulated pyroptosis. As an apoptotic initiator protease, *CASP9* is involved in tumor process, and Kim et al. (Kim et al., 2015) considered *CASP9* as a therapeutic target for treating cancer. According to our analytical results, *CASP4*, *CASP5*, and *CASP9* were upregulated in the tumor tissues, and their high expression indicated poor survival rate. These results suggested that they played a role as tumor-promoting genes in this study.

*ELANE*, a protease encapsulated in the main particle of the neutrophil precursor, could activate pro-inflammatory cytokines, comprising *TNF-*α, *IL-1*β, and *IL-18* (Fu et al., 2020). Kambara et al. (Kambara et al., 2018) demonstrated that GSDMD was under the cleavage and activation of ELANE for inducing pyroptosis of neutrophils. The more highly expressed ELANE was seen in the high-risk group than the low-risk group, while the neutrophil infiltration scores were apparently lower versus the low-risk group. These results may be due to the activation of the neutrophil focal death by *ELANE*.

*NOD1* belongs to the NOD-like receptor family (Correa et al., 2012). *NOD1* mutations are closely related to inflammatory diseases in humans, and a close relationship exists between inflammation and tumor (Layunta et al., 2018). Previous studies have confirmed that single nucleotide polymorphism of *NOD1* affects the occurring and advancement of various tumors, comprising lung carcinoma, stomach carcinoma, colorectal carcinoma, pancreatic carcinoma, head and neck squamous cellular carcinoma, etc (Cotterchio et al., 2015; Ozbayer et al., 2015; Suarez et al., 2015).

*NLRP3* is one of the most characteristic proteins in the inflammatory bodies of the NLR protein family, which has been proved to have relation with the occurring and advancement of carcinoma. *NLRP3* inflammosomes enhance the differentiation of gastric cancer cells by participating in *cyclin-D1* and inducing *IL-1*β production (Hai et al., 2016). In HCC, the molecular platform components of the *NLRP3* inflammasome are lost or significantly reduced compared with normal liver (Wei et al., 2014). We found that *NLRP3* was upregulated in the LUSC tissues and negatively correlated with survival time. *NLRP3* inflammasome could mediate pyroptosis through the cleavage of the GSDM family proteins, and *GSDMD* is one of main substrate of *NLRP3* inflammasome-induced pyroptosis (Martinon et al., 2004). The outcomes revealed the possibility of *NLRP3* inflammasome-induced pyroptosis in causing the development of some tumors.

*GPX4*, a member of the GPX family, is an important peroxide inhibitor protein. More and more explorations showed that *GPX4* is involved in the tumorigenesis. Reports have shown that *GPX4* expression is obviously higher in the liver biopsy tissue among patients suffering liver cancer than in nontumor tissue (Guerriero et al., 2015). Zhao et al. (Zhao et al., 2017) proposed that *GPX4* protein levels are high in glioma tissues and cell lines, and *GPX 4* has close relation with the proliferation, migration, and apoptosis of glioma cells. Explorations have shown that conditional *GPX4* knockout in monocytes promotes *CASP11* activation and *GSDMD*-mediated pyroptosis (Corrales et al., 2016).

In tumors, *IL-1B* is produced and secreted by a variety of cell types, like immune cells, fibroblasts, or carcinoma cells. As a proinflammatory cytokine that is expressed in primary tumors, *IL-1B* has been identified as a potential biomarker in patients with breast carcinoma (Martínez-Reza et al., 2019), *IL-1B* is highly expressed in the blood of patients with NSCLC (Kim et al., 2013), which was in consistency with the outcomes. High *IL-1B* levels had association with less overall and survival rates in LUSC patients. In conclusion, these nine genes have been confirmed to be related genes involved in tumorigenesis and development in the prognostic model. However, the manner by which these genes interact with each other in the process of pyroptosis remains to be further studied.

To date, full studies have not been performed on pyroptosis, hough we had known some resemblances in apoptosis and some crossover in the mechanism. With the advancement of tumors develop, coexistence and interaction might be seen in various patterns of cellular deaths (Fritsch et al., 2019). For example, in our model, three genes (i.e., *CASP4*, *CASP5*, and *CASP9*) were also identified as key regulators of the apoptotic pathway. Then, we analyzed the DEGs in the various risk groups and revealed that the DEGs got main involvement in leukocyte migration, regulation of immune effector process, and cytokine-cytokine receptor interaction, indicating that dead cells induced an intense inflammation response. Upon the basis of the GO and KEGG analysis, pyroptosis may be inferred to regulate the constitution of tumor immunization micro environment.

### 4.2 Correlation Between Pyroptosis and Tumor Immune Microenvironment

According to our results, except for nature killer (NK) cells, the infiltratory level of immune cells in the high-risk subgroup was normally lower contrast with the low-risk subgroup, suggesting an overall impairment of immunity functionalities in the high-risk group. Our study found the higher proportion of Treg cells in the low-risk group versus in the high-risk group. Treg cells are highly immunosuppressive, and in malignant tumors, these cells promote tumor progression by inhibiting effective antitumor immunity. High Treg infiltration has been observed in tumor tissues (Wolf et al., 2005), and the increase in the number of Treg and the decrease in the ratio of Treg have been associated with poor tumor prognosis (Toker et al., 2018). One likely cause for such difference may be that Treg cells are necessary in regulating the overactive inflammation response resulting from pyroptosis in the tumor microenvironment. In addition, all those 13 immune-associated pathways presented lower activity in the high-risk group versus the low-risk group. Upon the basis of such findings, the low surviving outcomes in high-risk LUSC patients may result from reduced levels of anti-tumor immunity.

At present, few studies have been conducted on pyroptosis in LUSC, especially its mechanism. preliminary exploration was made on the prognosis of such pyroptosis-related genes, providing theory foundation for upcoming explorations. Nevertheless, because of the deficiency in data, we can’t determine whether these genes also play a corresponding role in the pyroptosis pathway of LUSC, and this phenomenon deserves further investigation.

## 5 Conclusion

In summary, pyroptosis in the LUSC tissues was closely related to LUSC, as most pyroptosis-related genes are expressed differently amid healthy tissues and LUSC tissues. In addition, the score generated based on the risk markers for the nine genes associated with pyroptosis served as an independent risk factor in prediction of OS. The difference between the two risk groups had association with tumor immunity. This exploration provides a novel genetic marker for prediction of prognosis in LUSC and provides an essential foundation for further study of the relation amid genes associated with pyroptosis and immunity in LUSC.

## Data Availability

The datasets presented in this study can be found in online repositories. The names of the repository/repositories and accession number(s) can be found in the article/Supplementary Material.

## Abbreviations

CASP, cysteine-aspartic proteases; DEG, differentially expressed genes; GO, Gene Ontolog; GSDM, Gasdermin; KEGG, Kyoto Encyclopedia of Genes and Genomes; LASSO, The least absolute shrinkage and selection operator; LUSC, Lung squamous cell carcinoma; NK, nature killer; OS, overall survival; PCA, principal component analysis; ROC, receiver operating characteristic; ssGSEA, single-sample gene set enrichment analysis; TCGA, The Cancer Genome Atlas.
